# Exploring the effect of time and sex in family and community violence from 2008 to 2014

**DOI:** 10.11606/S1518-8787.2019053000910

**Published:** 2019-04-26

**Authors:** Kevan Guilherme Nóbrega Barbosa, Lorrany Gabriela Rodrigues, Gizelton Pereira Alencar, Sérgio D’avila, Efigênia Ferreira e Ferreira, Raquel Conceição Ferreira

**Affiliations:** I Universidade Federal de Minas Gerais. Faculdade de Odontologia. Belo Horizonte, MG, Brasil; II Universidade de São Paulo. Faculdade de Saúde Pública. São Paulo, SP, Brasil; III Universidade Estadual da Paraíba. Departamento de Odontologia. Campina Grande, PB, Brasil

**Keywords:** Violence, Domestic Violence, Violence Against Women, Physical Abuse, Time Series Studies

## Abstract

**OBJECTIVE:**

To evaluate the effect of the time and the sex of victims and perpetrators on the rates of family and community physical violence in a Brazilian municipality over seven years (2008–2014).

**METHODS:**

We made a census analysis from non-fatal victims attended in the Forensic Institute of the Scientific Civil Police. The monthly and annual violence rates were calculated based on the population size of the municipality. Time series was evaluated by negative binomial regression models, based on the number of cases with population offset and considering the effect of the sex of victims and perpetrators.

**RESULTS:**

A total of 3,324 cases of family and 4,634 cases of community violence were analyzed. There was a significant increase in family violence rates for female victims and male perpetrators. Family violence rates were always higher for female victims than for male and it was always lower for female perpetrators than for male (p < 0.001). There was a lower risk of community violence for male victims after 2013 and a decrease of aggression perpetrated by men over time. Men and women were similarly affected by community violence; however, the perpetrators were more frequently men.

**CONCLUSIONS:**

The results indicate a trend of increasing female victims in the family violence, mainly perpetrated by men. The reduction in community violence rates could be the result of policies to reduce crime.

## INTRODUCTION

Considering the recent recommendation of the 2030 Agenda for Sustainable Development, violence is one of the most urgent worldwide problems to be assessed in the next two decades^1^ ^,^ ^2^. Interpersonal violence (IPV) includes violent acts and intimidation between family members, intimate partners, or individuals who are known or unknown to each other^3^. This definition was presented in the Report on Violence and Health of the World Health Organization (WHO)^4^, which classified IPV into family violence (when involving intimate partners and family members) or community violence (when involving people who may be known or unknown to each other). The WHO report^4^ also distinguishes four modes in which violence may be inflicted (physical, sexual, psychological, and deprivation or neglect).

Every year, 1.3 million people die as a result of violence, accounting for 2.5% of world mortality; also, for individuals aged 15–44, violence represents the fourth global cause of death^5^. However, mortality represents only the “tip of the iceberg” because nonfatal violence is responsible for a less visible and more significant fraction^6^. For each death, there are innumerable other individuals who seek help due to injuries resulting from IPV^4^. A representative study carried out in the 26 Brazilian states and the Federal District revealed that 91.4% of all emergency department visits were related to aggression resulting from IPV^7^.

It is well recognized that the burden of IPV falls mainly on women, and this is a worldwide reality. If, on the one hand, 82% of homicide victims worldwide are male, the physical, sexual and psychological abuses mainly fall on women, especially in the family environment^5^. Family violence is very connected to affective and relational issues, especially when it involves intimate partners, whereas community violence occurs mainly in the social and economic context. The temporal evaluation of family and community violence rates, separately, considering the sex of victim and perpetrator, can contribute to understanding the pattern of occurrence according to the violence types and guide actions to prevent this ongoing process, considering the specificities involved in each type of violence^8^.

In this context, the aim of this study was to evaluate the trends in physical violence rates between January 2008 and December 2014 (84 months), considering the time and the sex of victims and perpetrators separately. The scenario used for data collection was an area located in the Northeastern region of Brazil.

## METHODS

### Data Source and Location

Data related to violence were obtained by consulting the records of traumatic physical offense reports in non-fatal victims from a Forensic Service in Campina Grande, PB, Brazil.

Campina Grande is a medium-sized city located in the central region of the state of Paraíba, at Northeastern Brazil, and represents an important link between the state capital (Atlantic coast) and other regions (west regions). Counting with a 402,912 population in 2016^a^, the municipality is a metropolitan region that influences surrounding cities, with intense commerce, industry and university activity.

The Forensic Service corresponds to the official service of the state government to carry out *corpus delicti* examinations on victims of physical violence. The examination is performed by an official expert, usually a physician and occasionally a dental surgeon (when it involves the face), who describes the visible wounds in victims and issues a traumatological report. The performance of *corpus delicti* examination is essential in legal proceedings against the accused of a crime, and can only be performed after an official referral of a police authority or demand for legal authority. This examination is legislated by Decree-Law 3,689^9^ of 1941 of the Brazilian Code of Criminal Procedure, which is a legal instrument in criminal trials and its absence may result in nullity of criminal proceedings.

We included all reports of IPV (only physical wounds or injuries) occurring in Campina Grande during the period of 84 months, between January 2008 and December 2014. The type was classified as family or community violence according to WHO (2002)^4^. The following variables were also considered in this study: a) year or month of occurrence; b) sex of victims (male or female); and c) sex of perpetrators (male or female). We did not include reports of sexual, psychological, and neglected violence.

Before data extraction, theoretical training was carried out aiming to standardize the information search process, with the participation of eight team members. The discussion considered how and where the variables in the study were present in the reports, following the WHO classification^4^ of family or community violence. Then, a pilot study was conducted among all team members, with 30 reports of 2007 (not included in the research), with the purpose of analyzing the information and preparing the final form. To test the inter-examiner differences, 30 reports from 2007 (not included in the research) were read in a two-week interval for each evaluator, and after that, the agreement was compared. The Kappa result between all eight researchers was satisfactory, ranging between 0.75 and 0.85.

### Statistical Analysis

Initially, the descriptive analysis of the type of violence was performed considering the percentage of cases throughout the years. Subsequently, the description of rates/100,000 population of IPV was conducted considering the time in months between 2008 and 2014, and the graphical representation was held by the Lowess (Locally Weighted Scatterplot Smoothing) method, based on the tricubic function. The monthly rates were analyzed to verify possible seasonality. First, the trend of the family and community rates for female/male victims and perpetrators was evaluated through the negative binomial regression model. The population offset for the municipality was based on the last Brazilian Census^b^ (53% female and 47% male) and the results of effects were presented as annual percentage variation. This annual variation was obtained by the exponential of the product of the regression coefficient by 12 months.

Four negative binomial regression models were used to evaluate: 1) the effect of the sex of victims and the time on family violence; 2) the effect of the sex of perpetrators and time on family violence; 3) the effect of sex of victims and time on community violence; and 4) the effect of sex of perpetrators and time on community violence. The incidence rate ratios (IRR) were estimated and the interaction between sex and time was tested on all four models in which the time was considered in months. Moreover, for each model, one dummy variable was included to test for possible trend changes at a certain month, defined by the visual inspection of the series. In this case, a new binary variable was created (0: before and 1: after the defined month). The binomial negative regression was compared to the Poisson regression model by the likelihood ratio test of the parameter alpha. The binomial negative model was used to account for overdispersion of data.

In all analyses, the significance level was 5%. Also, the Stata program (version 14.2 for Windows) was used to compute the regression model and the graphs.

### Ethical Statement

The study follows Brazilian standards for human research (Resolution 466/12 of the Ministry of Health) and the international standards of the Declaration of Helsinki. It was approved by the Research Ethics Committee of the Universidade Estadual da Paraíba (Protocol 0652.0.133,000-11) and the Universidade Federal de Minas Gerais (Protocol 47207815.5.0000.5149). Furthermore, we declare no conflict of interest in the stages of this research, including data extraction and manuscript elaboration.

## RESULTS

A total of 8,270 records of IPV were searched, including 4,634 cases of community violence, 3,324 cases of family violence, and 312 records (3.7%) that had no information about the date of occurrence.

Table 1 summarizes the descriptive analysis of the annual series about the type of violence considering the relationship between victim-perpetrator. The major variation in family violence occurred between intimate partners; increasing from 10.6% (2008) to 16.8% (2014). In community violence, the major variation involved violence practiced by an unknown person, with a negative decrease of 14.2% between 2008 and 2014. Among events involving intimate partners, women were victims in 88.5% of the cases; in community violence, 80.2% of male victims suffered aggression by an unknown person. The other frequencies for the female sex were: ex-partner (82.6%); family member (66.6%), and known person (58.5%).

**Table 1:** Frequency of the relationship victim-perpetrator and rates per 100,000 population according to the type of violence over time. (n = 7,921)^a^

Variable	2008	2009	2010	2011	2012	2013	2014	Total	Δ^b^ (Delta)
Family									
Partner	160 (10.6%)	149 (9.9%)	179 (11.9%)	210 (14.0%)	258 (17.2%)	296 (19.7%)	252 (16.8%)	1,504 (100.0%)	+6.2%
Ex-partner	142 (16.0%)	105 (11.9%)	107 (12.1%)	141 (15.9%)	117 (13.2%)	131 (14.8%)	143 (16.1%)	886 (100.0%)	+0.1%
Family	151 (12.1%)	134 (10.7%)	115 (9.2%)	203 (16.2%)	228 (18.2%)	208 (16.6%)	214 (17.1%)	1,253 (100.0%)	+5.0%
Total	453 (12.5%)	388 (10.7%)	392 (10.8%)	554 (15.2%)	603 (16.5%)	635 (17.5%)	609 (16.8%)	3,634 (100.0%)	+4,3%
Rate/100,000 population	6.8	6.3	8.7	10.1	12.7	13.4	12.6	10.1	
Community									
Known-person	461 (15.3%)	382 (12.6%)	426 (14.1%)	467 (15.5%)	442 (14.6%)	459 (15.2%)	383 (12.7%)	3,020 (100.0%)	-2.6%
Unknown-person	259 20.6%	171 13.6%	302 24.0%	236 18.8%	112 8.9%	98 7.8%	80 6.4%	1,258 (100.0%)	-14.2%
Total	720 (16.8%)	553 (13.0%)	728 (17.0%)	703 (16.4%)	554 (13.0%)	557 (13.0%)	463 (10.8%)	4,278 (100.0%)	-6.0%
Rate/100,000 population	18.8	14.4	15.2	17.8	12.0	11.5	9.6	14.2	
Total	1,173 (14.8%)	941 (11.9%)	1,129 (14.3%)	1,257 (15.9%)	1,157 (14.6%)	1,192 (15.0%)	1,072 (13.5%)	7,921 (100.0%)	

^a^ Missing information excluded (n = 349).

^b^ Variation of the percentage between 2014 and 2008.

**Figure 1:**
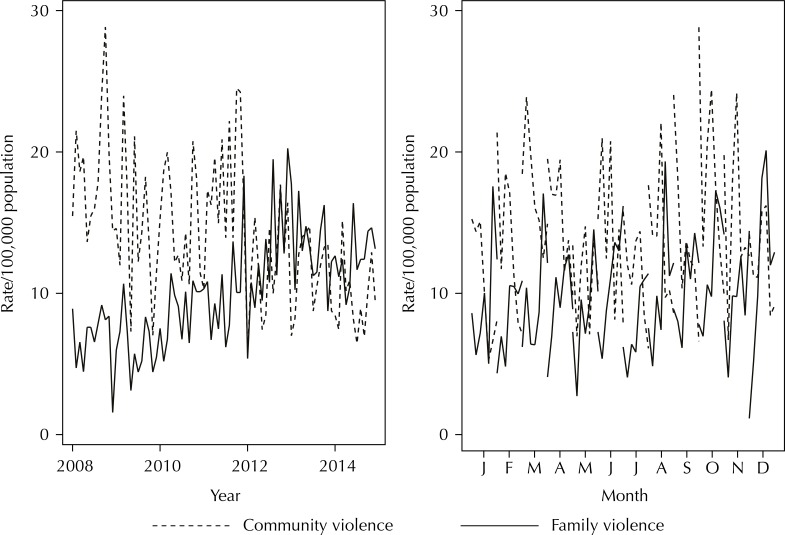
Monthly and annual time series of community and family violence rates.

There was an increase in family violence rates and a reduction in community violence over seven years (Figure 1). The distribution of rates, from 2008 to 2014, was similar over the months, indicating no seasonality. Higher IPV rates were observed in March and October (Figure 1).

**Figure 2:**
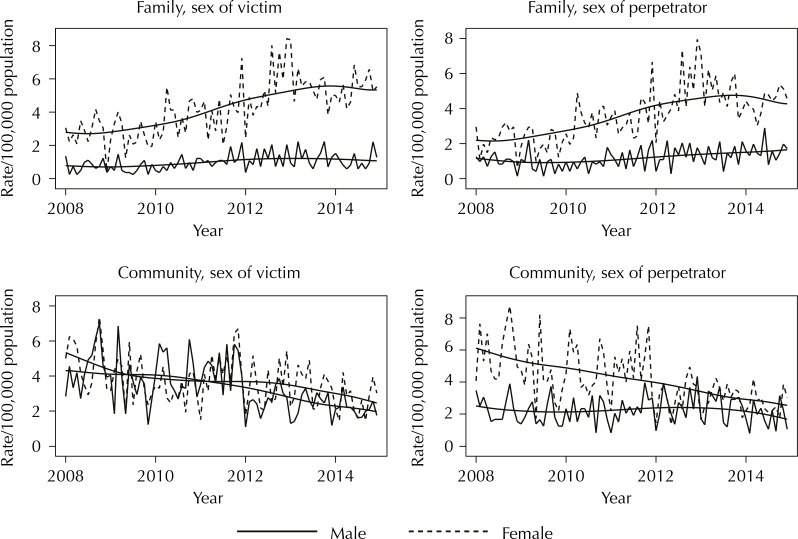
Time series of family and community violence by sex of the victim and sex of the perpetrator.

The analysis of family violence showed that the annual average rate of general family violence was 6.8/100,000 in 2008, increasing to 12.6/100,000 in 2014 (Table 1). There was a significant increase in the family violence rate over time involving female victims (β = 0.011; 95%CI 0.007–0.016; annual percentage variation: +14.8%; 95%CI +8.8%, +21.0%; p < 0.001), and male perpetrators (β = 0.012; 95%CI 0.007–0.017; annual variation: +15.3%; 95%CI +8.9%, +22.0%; p < 0.001). There was no significant time effect on the family violence rate involving male victims (β = 0.009; 95%CI -0.0004–0.018; annual percentage variation: +10.9%; 95%CI -0.8%, +23.5%; p = 0.06) and female perpetrator (β = 0.008; 95%CI -0.0002–0.017; annual variation: +10.5%; 95%CI +0.1%, +22.0%; p = 0.05) (Figure 2).

In regression models, the interaction coefficients represent the slopes of the outcome on the time for each of two levels of sex. Model 1 and 2 showed a significant increase in family violence rates for female victims and male perpetrators, therefore, the average risk with the time was 1.01 for both (p < 0.001). The main effect of sex was significant in the two models, which indicates that family violence rates were always higher for female victims than for male (p < 0.001) and that it was always lower for female perpetrators than for male (p < 0.001) (Table 2).

**Table 2:** Effect of time, sex of victims and sex of perpetrators on family and community violence. Campina Grande, state of Paraíba, Brazil, Jan. 2008 to Dec. 2014.

Type of violence	Models	Variable	IRR	95%CI	p
Family	Model 1 for victims^a^	Sex of victim			
		Male	1	1	
		Female	3.39	2.17–6.31	**< 0.001**
		Sex of victim×time (months)			
		Male	1.00	0.9–1.02	0.060
		Female	1.01	1.01–1.02	**< 0.001**
	Model 2 for perpetrators^a^	Sex of perpetrator			
		Male	1	1	
		Female	0.38	0.23–0.64	**< 0.001**
		Sex of perpetrator×time (months)			
		Male	1.01	1.00–1.02	**< 0.001**
		Female	1.00	0.99–1.02	0.05
Community	Model 3 for victims	Sex of victim			
		Male	1		
		Female	1.05	0.54–2.04	0.882
		Sex of victim×time (months)			
		Male	0.99	0.99–1.01	0.978
		Female	0.99	0.98–1.00	0.286
		Sex of victim×dummy time (Jan 2013)			
		Before, Male	1		
		Before, Female	1.05	0.67–1.63	0.831
		After, Male	0.58	0.37–0.92	**0.020**
		After, Female	^b^		
	Model 4 for perpetrators^a^	Sex of perpetrator			
		Male	1		
		Female	0.36	0.27–0.51	**< 0.001**
		Sex of perpetrator×time (months)			
		Male	0.99	0.98–0.99	**< 0.001**
		Female	1.00	0.99–1.01	0.947

IRR: incidence rate ratios

^a^ In model 1, 2 and 4, the dummy variable was not significant (p > 0.05).

^b^ Omitted due to collinearity.

Statistically significant results are shown in bold.

The analysis of the community violence showed that the average rate of general community violence decreased from 18.8/100,000 in 2008 to 9.6/100,000 in 2014 (Table 1). There was a significant reduction in the community violence rate involving male victims (β = -0.009; 95%CI -0.014– -0.004; annual percentage variation: -10.5%; 95%CI -15.5– -5.11; p < 0.001), and female victims (β = -0.006, 95%CI -0.010– -0.001; annual percentage variation: -6.6%; 95%CI -11.6– -1.25; p = 0.016). There was a significant reduction in the community violence rate for male perpetrators (β = -0.009, 95%CI -0.014– -0.005; annual percentage variation: -11.1%; 95%C -15.7– -6.3; p < 0.001). There was no significant effect of time on the community violence rates by female perpetrators (β = 0.0002, 95%CI -0.006–0.006; annual percentage variation: +0.2%; 95%CI -6.6– +7.6, p = 0.947) (Figure 2).

Considering community violence rates, there was a significant interaction between sex (victim and perpetrator) and time. A dummy variable (time cut in 48 months) was included in model 3. This model showed a lower risk of community violence over time (comparing after and before 48 months) for male victims. The main effect of the sex of victim was not significant for community violence rates. Model 4 showed that the main effect of sex of perpetrator was significant, which indicates that the community violence rates perpetrated by women were lower over time (p < 0.001). In the same model, a significant decrease was observed in the community violence perpetrated by men and a rate that remains constant over time was obtained for female perpetrators (Figure 2).

**Figure 3:**
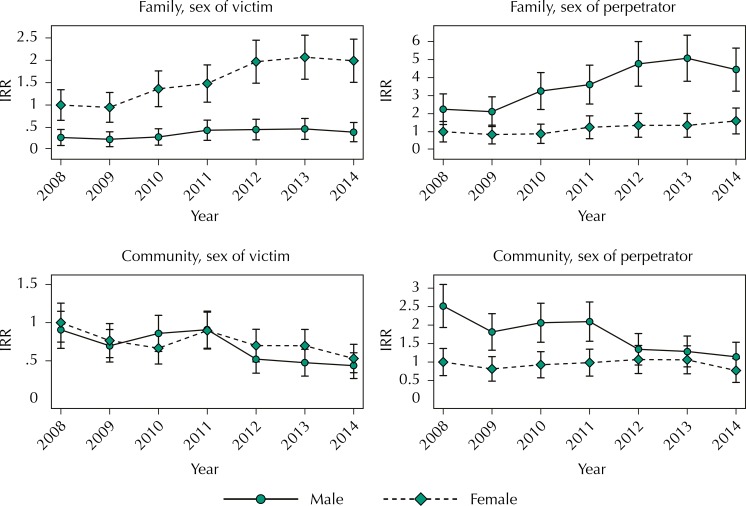
Adjusted predictors (IRR) of interaction time *versus* sex with 95%CI.

Regarding the interaction between sex (victim and perpetrator) and time, the IRR for each year and sex (victim and perpetrator) was presented considering as a reference: 2008 and female sex (Figure 3). The figures showed the IRR changes over time.

IRR: incidence rate ratios

## DISCUSSION

This study described the evolution of IPV rates in a Brazilian area. In the family context, the violence over time happened mainly among intimate partners and were perpetrated by men, with women being the predominant victims. The temporal analysis showed an increase in the rates of family violence for female victims and male perpetrators. There was a lower risk of community violence for male victims after 2013 and a decrease of cases perpetrated by men over time. Men and women were similarly victims of community violence and male perpetrators were more frequent. The drop in the community violence was mainly related to the decrease in violence perpetrated by an unknown person, since the known-perpetrator component remained high and practically unchanged over the seven years. In this study, the known-perpetrator component typically included friends, co-workers, or someone in the community, while unknown-perpetrator component usually included muggers or those involved in fights and discussions at nightclubs and bars.

High prevalence of physical violence in women occurred in other regions of Brazil, as demonstrated by Schraiber et al.^10^ using data that were part of the classic multi-center study conducted by WHO on violence against women. Historically, the violence against women represents a serious social problem that directly affects several countries; however, the estimate is that 20%–60% of women do not report the violence suffered^5^. The increase in female victims is contrary to the federal government’s attempt to stop the advance of abuses committed against women, considering that the 2006 National Law 11.340^11^ was issued with the aim of decreasing domestic violence. However, the Paraíba State Report^c^ on violence argues that this increase may be apparent, and is likely related to a greater dissemination of the importance in denouncing cases of violence and to the increase of women’s empowerment to seek support in assistance networks.

Historically, the Americas have always had a high prevalence of violence against women, being surpassed only by some regions of the Middle East and Africa^12^. Even in the United States, America’s most developed country, CDC^13^ research data found that approximately 32.9% percent of U.S. women experienced physical violence in their lives, and about one in three women have suffered some slap or push from their intimate partners. On the other hand, Canada^14^ has successfully pursued policies to reduce violence against women, since prevention and intervention initiatives for family violence have begun to be implemented since 1988. In Latin America, a study conducted in three large metropolises in Brazil, Chile, and Mexico reported a lifetime prevalence of physical violence against women of 27.9%, 31.4%, and 30.7%, respectively^15^.

The higher rate of family violence perpetrated by men over time points to something already expected and reported: men are more violent. Several world reports of the WHO (2002^4^, 2010^16^, 2014^5^) have confirmed this as a worldwide condition^17^. It is likely that social and biological aspects play a significant role in this context, and this duality can be used as an attempt to understand why men are more likely to assault women. Batrinos^18^ in a review article explained that men, from a biological perspective, remain with some atavistic levels of testosterone that can be manifested in different ways, from sports activities and competition to dominating behavior and physical violence. In addition, there is the social component, which for centuries has accepted physical violence, especially against women and children, as a normal rule in the domestic environment^19^.

Aggressive behaviors that men practice against women are usually more serious; however, there is evidence contrary to our findings that women are also responsible for a growing share of aggression to intimate partners^20^. A population-based study conducted in India showed that men reported a higher life-prevalence for physical aggression by their partner, while sexual violence fell more on women^21^. Flood and Pease^22^ argued that, in the case of violence against women, an important domain for perpetuating assaults is by male sexist attitudes and contempt for women. Another important issue, although not available for analysis in the present research, corresponds to family income. Studies carried out in large metropolitan areas in the United States have found that women living in poverty and poor communities tend to report more physical violence and are more victims of murder^23^ ^,^ ^24^.

In Brazil, efforts against domestic violence toward women had some advances that have tried to increase reporting, supervision, and punishment of violent events as Law 10,778/2003^25^, Law 11,340/2006^26^, Law 13,104/2015^27^, and some actions such as the creation of the Victim Assistance Center; centers and assistance networks for women who have been raped; and the “Woman: Living without Violence” program. Despite government actions and the feminist sector in the fight against violence, national data^28^ have shown that violence against women is increasing in the country, as discussed in the current study about family violence.

The findings for community violence, especially when involving unknown perpetrators, may indicate the contribution of some governmental actions. The reduction in community violence rates after 2012 coincided with the change of government, which instituted a program^29^ to fight violence, starting in 2011. This program involved the participation of society, Public Ministry, and Judiciary Power and aimed to reduce crime in the state through prevention, ostensible, and qualified repression actions, such as intelligence work. Government actions alone cannot stop community violence; the extinction of the drug market and intensive policing at crime hotspots reduced criminality rate in the United States, especially in New York City, from 2,245 homicides in 1990 to 328 in 2014^30^. Nevertheless, there is evidence that homicidal violence and aggression have increased in large U.S. cities between 2015 and 2016, forcing the government to adopt federal strategies in these places^31^. Another example of violence reduction was achieved in Honduras after a three-year community-based approach focused on citizenship activities and social-community participation^32^.

Federal attempts to suppress community violence in Brazil had great results since the implementation of the National Policy on Morbidity and Mortality by Accidents and Violence in 2001, improving medical and hospital care for victims of violence^33^. In 2003, the National Law 10,826^34^ increased the prohibition of firearm possession by the civilian population, which caused a visible drop in homicide rates in subsequent years; however, this rate has increased again since 2007^35^. This situation reinforces the fact that government actions alone do not change community violence in the long term. In this context, the involvement of the sectors of civil society in facing the problem is also necessary.

No seasonality was observed in series; however, there was a peak in community violence rates in the months of March and October. Those peaks are probably related to some local characteristics as the Campina Grande city has its economy mainly supported by commercial, university or school activities, and social events during the equinox months (March to June, and September to November). In summer and winter, the city suffers a “population drain” due to school and university holidays and many families travel to more inland cities. Interestingly, in June, the month of the greatest festive event in the region (30 days of celebrations), there was a lower incidence of violence, probably due to a greater community policing effort or a possible underreporting in the domestic environment.

Study limitations such as underreporting, especially in cases of family violence involving intimate partners and the non-use of hospital data to compute violence rates, must be considered when analyzing the real magnitude of violence. Certainly, violence rates are even higher than those observed; however, official data from the state government were used to assess IPV, which *a priori* holds the greatest amount of information regarding physical violence.

The results presented are crucial for decision-making and public safety managers and suggest that actions to control the increase of domestic violence against women are urgently needed in the face of the significant increase of victims over the years. Measures to control community violence must be continuously encouraged regardless of government changes, in the face of a visible successful experience of reducing violence rates over the years.

In the family environment, the results indicate a tendency of worsening the number of physical violence against women, and a growing increase of aggression perpetrated by men. Partially, these results could be related to increased reporting and women’s empowerment to denounce. According to our findings, federal attempts to reduce violence against women do not seem to have reached the expected effect for the study region, which reinforces the need for a more careful look at the problem, both by the government and civil society. In contrast, the results point to a successful experience with community violence, whose rates suggest a decreasing trend over the years. It is likely that government efforts, such as programs to fight crime, have contributed to this situation. Finally, it must be highlighted the importance of carrying out temporal monitoring of violence as a useful tool to predict the trend of this phenomenon over time, guiding decision-making and actions on factors that exhibit increasing behavior (such as family violence), and as a means of monitoring declining factors (such as community violence).

## Footnotes

a Instituto Brasileiro de Geografia e Estatísticas. Downloads: estatísticas. Rio de Janeiro: IBGE; 2017 [cited 2017 Apr 18]. Available from: http://downloads.ibge.gov.br/downloads_estatisticas.htm

b Instituto Brasileiro de Geografia e Estatísticas. Censo 2010. Rio de Janeiro: IBGE; 2010 [cited 2017 Apr 18]. Available from: http://downloads.ibge.gov.br/downloads_estatisticas.htm

c Governo do Estado da Paraíba, Secretaria da Saúde. Violência contra a mulher: relatório. João Pessoa, PB; 2012.
